# Impact of Hyperkalemia in Heart Failure and Reduced Ejection Fraction: A Retrospective Study

**DOI:** 10.3390/jcm12103595

**Published:** 2023-05-22

**Authors:** Andrea Lopez-López, Raúl Franco-Gutiérrez, Alberto José Pérez-Pérez, Margarita Regueiro-Abel, Juliana Elices-Teja, Charigan Abou-Jokh-Casas, Carlos González-Juanatey

**Affiliations:** 1 Cardiology Department, Hospital Universitario Lucus Augusti, 27003 Lugo, Spain; andrea.lopez.lopez@sergas.es (A.L.-L.); raul.franco.gutierrez@sergas.es (R.F.-G.); alberto.jose.perez.perez@sergas.es (A.J.P.-P.); margarita.regueiro.abel@sergas.es (M.R.-A.); juliana.elices.teja@sergas.es (J.E.-T.); charigan.abou.jokh.casas@sergas.es (C.A.-J.-C.); 2 Biodiscovery HULA-USC Group, Instituto de Investigación Sanitaria de Santiago de Compostela IDIS, 27003 Lugo, Spain

**Keywords:** hyperkalemia, systolic heart failure, mortality

## Abstract

(1) Background: Hyperkalemia is a common finding in patients with heart failure and reduced ejection fraction (HFrEF), though its prognostic significance is controversial. There is no consensus on optimal potassium levels in these patients. The primary endpoint of this study was to determine the 5-year incidence of hyperkalemia in a cohort of patients with HFrEF. Secondary endpoints were to determine predictors of hyperkalemia and its impact on overall 5-year mortality; (2) Methods: retrospective, longitudinal, single-center observational study of patients with HFrEF followed-up in a specialized unit between 2011 and 2019. Hyperkalemia was considered as potassium concentration > 5.5 mEq/L; (3) Results: Hyperkalemia was observed in 170 (16.8%) of the 1013 patients. The 5-year hyperkalemia-free survival rate was 82.1%. Hyperkalemia was more frequent at the beginning of follow-up. Factors associated with hyperkalemia in the multivariate analysis were baseline potassium (HR 3.13, 95%CI 2.15–4.60; *p <* 0.001), creatinine clearance (HR 0.99, 95%CI 0.98–0.99; *p* = 0.013), right ventricular function (HR 0.95, 95%CI 0.91–0.99; *p* = 0.016) and diabetes mellitus (HR 1.40, 95%CI 1.01–1.96; *p* = 0.047). The overall survival rate at 5 years was 76.4%. Normal–high potassium levels (5–5.5 mEq/L) were inversely associated with mortality (HR 0.60, 95%CI 0.38–0.94; *p* = 0.025); (4) Conclusions: Hyperkalemia is a common finding in patients with HFrEF with an impact on the optimization of neurohormonal treatment. In our retrospective study, potassium levels in the normal–high range seem to be safe and are not associated with increased mortality.

## 1. Introduction

Potassium is the most common cation in the human body and is essential for proper cellular function [1]. Hyperkalemia, defined as an increase in plasma potassium concentration exceeding 5.0 mEq/L, is associated with alterations in cell action potential and in cardiac conduction and excitability [2] that can result in an increased mortality risk [3,4,5,6].

The prevalence of hyperkalemia in patients admitted to hospital ranges between 1 and 10% [7], with its occurrence being 2–3 times more likely in patients with heart failure [8,9], mainly due to the presence of comorbidities that promote a water–electrolyte imbalance (such as diabetes mellitus or chronic kidney disease), and also due to neurohormonal treatment [10].

The use of angiotensin receptor neprilysin inhibitors (ARNIs), angiotensin converting enzyme inhibitors (ACEIs) or angiotensin II receptors blockers (ARBs), together with mineralocorticoid receptor antagonists (MRAs), has improved survival and has diminished hospital admission rates in patients with heart failure and reduced left ventricular ejection fraction (HFrEF) [11]. However, these drugs have also been associated with an increased risk of high potassium levels. In a recent study, the presence of hyperkalemia prevented the achievement of target doses in up to 4.6% of the patients treated with ACEIs or ARBs and in 15% of those treated with MRAs, and was responsible for 14.2% and 33.3% of the contraindications to ACEI/ARBs and MRAs, respectively [12]. The need for drug interruption or dose reduction, particularly when reaching less than half of the recommended dose of renin–angiotensin system inhibitors, results in a loss of the prognostic benefits of these drugs compared with individuals in which full doses are reached [13,14].

The relationship between plasma potassium levels and mortality in patients with heart failure is contradictory [15,16]. While low potassium concentrations have been consistently associated with increased mortality [17,18], available data referring to hyperkalemia are heterogeneous [3,6,15,16,19]; thus, there is no consensus regarding the optimum or maximum potassium levels allowed in patients with HFrEF [15]. Furthermore, little information is available on the serum potassium levels and their real-life prognostic implications in these patients. In this context, the need arises to know both the percentage of individuals with significant hyperkalemia and to assess the long-term prognostic impact of potassium levels in a cohort of patients with HFrEF.

The primary objective of the study was to analyze the incidence of hyperkalemia in a cohort of patients with HFrEF subjected to follow-up in a specialized heart failure unit. The secondary objectives were to identify the clinical profile of patients diagnosed with hyperkalemia and the potential impact of the latter upon mortality over a 5-year follow-up period.

## 2. Materials and Methods

### 2.1. Study Design and Patients

A retrospective, longitudinal, single-center observational study was conducted of consecutive patients starting follow-up in the Specialized Heart Failure Unit of the Department of Cardiology of a secondary hospital between January 2011 and December 2019.

The study included patients with HFrEF defined according to the clinical practice guidelines from the European Society of Cardiology (ESC) of 2021 [20]. The subjects were all Caucasian, over 18 years of age and were referred from the hospital admission unit or from other cardiology clinics. None of them were in acute heart failure decompensation.

### 2.2. Data Collection

Demographic, clinical, laboratory testing, electrocardiographic and echocardiographic data, as well as the information concerning treatment and successive controls, were collected by the cardiologists assigned to the unit, based on reviews of the electronic case histories of the patients. The variables were recorded and stored in a pre-existing database that was specifically designed for this particular study.

Ejection fraction (at baseline and during follow-up) was evaluated by echocardiography (IE-33—Phillips Netherlands and EPIQ-5—Phillips Netherlands).

Patient management abided by the heart failure guides applicable at the time of patient assessment in the clinic.

Hyperkalemia was defined as a serum potassium concentration over 5.5 mEq/L. The plasma potassium levels were obtained before each patient’s visit (no measurement was recorded during the patient’s hospital admission). The presence of hyperkalemia was assumed at the first measurement that met the diagnostic criterion. 

Mortality was defined as overall mortality. Follow-up started on the date of the first visit to the unit and ended after 5 years.

### 2.3. Ethical Aspects

The study was carried out following the Good Clinical Practice (GCP) guidelines, ethical principles, and the Spanish legal specifications regarding research in force at the time of the study. Approval was obtained from the local research ethics committee before starting the study.

### 2.4. Statistical Analysis

Quantitative variables were reported as means with their standard deviation (SD), and qualitative variables were reported as frequencies and percentages. The chi-squared test, or Fisher’s exact test where the former was not possible, was used to compare qualitative variables, while the Student *t*-test was applied for quantitative variables.

The uni- and multivariate assessment of the mortality predictors were based on Cox regression analysis. The event-free survival curves were generated using the Kaplan–Meier method.

Statistical significance was considered for *p* < 0.05.

All statistical analyses were performed with IBM-SPSS software v19 (IBM, Armonk, NY, USA).

## 3. Results

### 3.1. Baseline Characteristics

A total of 1013 patients met the inclusion criteria. The baseline characteristics of the subjects are summarized in Table 1.

### 3.2. Incidence and Predictors of Hyperkelemia

**Figure 1:**
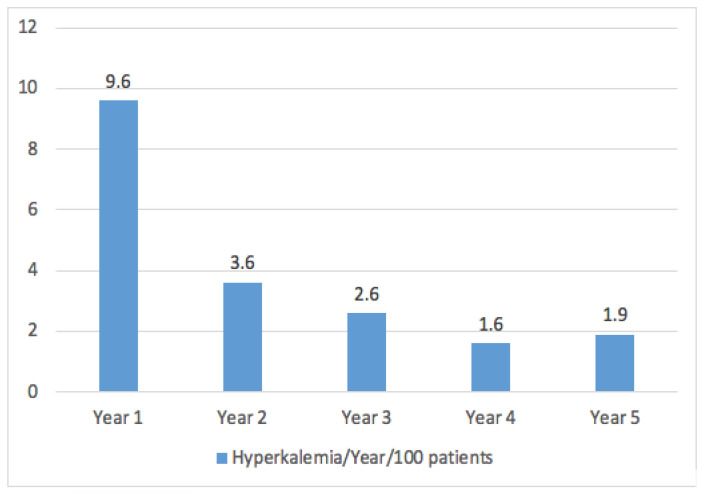
First hyperkalemia event per year and 100 patients.

**Figure 2:**
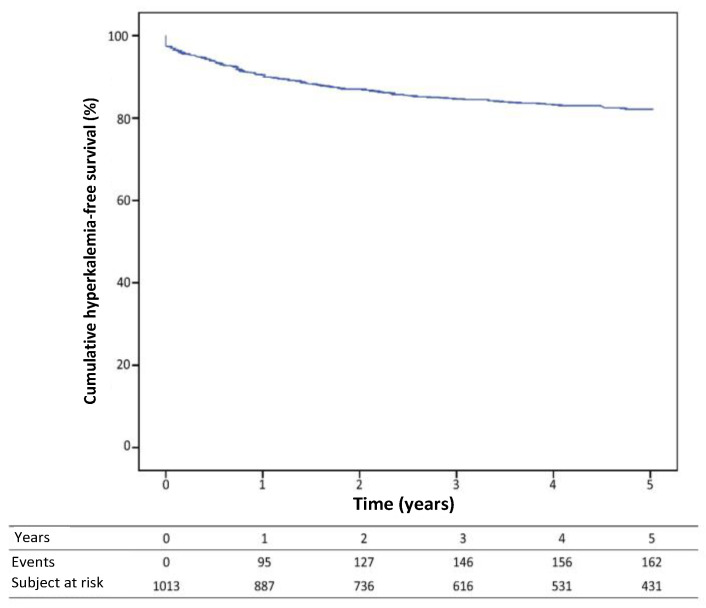
Kaplan–Meier hyperkalemia-free survival curve.

In the cohort of patients under study, a vast majority of 97.7% (n = 990) had their potassium levels assessed during the week preceding their medical consultation. It is noteworthy that none of the patients were in a state of acute decompensation of heart failure, and no measurements were taken during such episodes or hospital admissions. Hyperkalemia was detected in 170 patients (16.8%) based on at least one measurement. The graphical representation in Figure 1 illustrates the trend of hyperkalemia probability over the course of the follow-up period as it relates to evolutionary changes. The study found that the occurrence of hyperkalemia was initially recorded at a rate of 9.5 cases per 100 patients during the first year, but exhibited a subsequent decline over time. The rate of survival without hyperkalemia at 1, 2 and 5 years was 90.5% (0.9), 87.0% (1.1) and 82.1% (1.3), respectively, as illustrated in Figure 2.

Considering the first year of follow-up, the hyperkalemia-free survival rate was 95.3% (0.7) at three months, 93.7% (0.8) at 6 months, 92.0% (0.9) at 9 months and 90.5% (0.9) at one year.

The univariate analysis identified the following hyperkalemia predictors (Table 2): age (*p* < 0.001), creatinine clearance (*p* < 0.001), QRS < 120 ms (*p* < 0.001), baseline potassium (*p* < 0.001), hematocrit (*p* = 0.001), diabetes mellitus (*p* < 0.001), end-diastolic left ventricular diameter (*p* = 0.032), pulmonary artery systolic pressure (*p* = 0.003), right ventricular function assessed by TAPSE (*p* = 0.006), the presence of mitral valve regurgitation (*p* = 0.005), diastolic blood pressure (*p* < 0.001), use of beta-blockers (*p* = 0.045) or loop diuretics (*p* = 0.021) and ischemic heart disease (*p* = 0.034).

The multivariate analysis identified the following hyperkalemia predictors (Table 3): baseline potassium (*p* < 0.001), creatinine clearance (*p* = 0.013), right ventricular function (*p* = 0.016) and diabetes mellitus (*p* = 0.047). The initial levels of potassium were recorded as 4.66 ± 0.44 mEq/L, while the creatinine clearance (MDRD) was observed to be 73.1 ± 24.6 mL/min/1.73 m^2^ as stated in Table 1.

### 3.3. Overall Survival and Mortality Predictors

**Figure 3:**
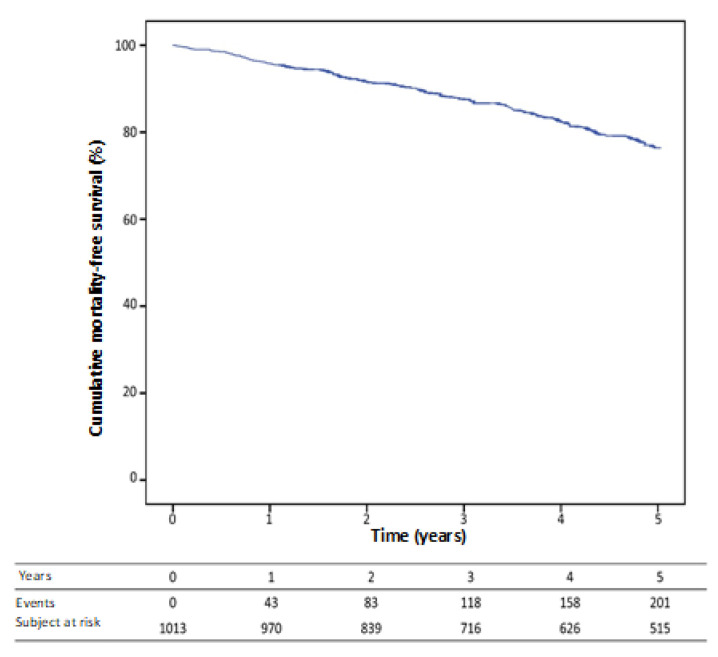
Kaplan Meier free survival curve.

Overall survival was 95.8% (0.6) at one year, 91.5% (0.9) at two years and 76.4% (1.5) at 5 years (Figure 3).

The univariate analysis identified the following mortality predictors (Table 4): female gender (*p* < 0.001), end-diastolic left ventricular diameter (*p* < 0.001), pulmonary artery systolic pressure (*p* < 0.001), right ventricular function (*p* < 0.001), arterial hypertension (*p* = 0.011), presence of diabetes mellitus (*p* < 0.001), NYHA functional class (*p* < 0.001), age (*p* < 0.001), smoking (*p* = 0.001), creatinine clearance (*p* < 0.001), the presence of ventricular arrhythmias (*p* < 0.001), baseline potassium (*p* < 0.001), baseline hematocrit (*p* = 0.001), systolic (*p* = 0.029) and diastolic blood pressure (*p* = 0.002), ACEI/ARB therapy (*p* < 0.001) and loop diuretics usage (*p* < 0.001), ischemic heart disease (*p* < 0.001), and implanted defibrillator and/or resynchronization therapy at the time of first assessment (*p* = 0.049).

The multivariate analysis identified the following mortality predictors (Table 5): age (*p* = 0.002), female gender (*p* < 0.001), presence of diabetes mellitus (*p* = 0.003), advanced NYHA functional class (*p* = 0.034), baseline hematocrit (*p* = 0.013), creatinine clearance ≥ 60 mL/min/1.73 m^2^ (*p* = 0.007), mild hyperkalemia (*p* = 0.025), right ventricular function (*p* = 0.012), the presence of ventricular arrhythmias (*p* < 0.001) and ACEI/ARB therapy (*p* = 0.020).

## 4. Discussion

In the present study, the prevalence of hyperkalemia in patients with HFrEF was found to be high, though normal–high potassium levels were found to exert a protective effect in relation to overall mortality.

The prevalence of baseline hyperkalemia in our study (2.4%) was lower than in previously published registries (4.3% [12] and 5.7% [21]). Since the baseline characteristics of the patients were similar in all three studies, the main observed differences may be explained by the higher percentage of individuals that received ACEI/ARB/ARNI (92% in the ESC-EORP-HFA Heart Failure Long Term study, 94% in the SPANIK-HF and 79% in our study) and MRA (73% versus 63.8% in the mentioned registries, and 58.4% in our sample), since these patients were under follow-up in specialized units in the drug titration phase [12] or had completed titration [21], while our patients were starting follow-up in such a unit for the first time. The high incidence of hyperkalemia in the first year of follow-up in our population, in comparison to subsequent years, could be explained by the fact that it is in the first months after patient referral when the progressive titration of neurohormonal treatment takes place. In our study, at one-year follow-up, 97.8% of patients were on ACE inhibitors/ARB/ARNI at higher doses than at baseline; 60.0% were receiving MRAs at doses similar to baseline; and a smaller proportion (53.9%) of subjects treated with diuretics were receiving lower doses than at baseline. Other factors that could have influenced the high percentage of hyperkalemia observed are the long duration of follow-up and the number of laboratory test measurements performed (97.7% of the visits versus 89.2% in the ESC-EORP-HFA registry).

The identified hyperkalemia predictors were baseline potassium, creatinine clearance, the presence of diabetes mellitus and right ventricular function. Both impaired renal function and diabetes mellitus have been associated classically with an increased risk of hyperkalemia [22,23]. Although potassium homeostasis is usually not affected until glomerular filtration rate falls below 10–15 mL/min [24], the administration of drugs that act upon the renin–angiotensin–aldosterone axis could facilitate the appearance of hyperkalemia in the presence of a mild to moderate decrease in the glomerular filtration rate [20]. The co-occurrence of diabetes mellitus and hyperkalemia can be elucidated by two mechanisms. Firstly, it is associated with hyporeninemic hypoaldosteronism, which results in the inability to regulate tubular potassium excretion [24]. Secondly, the insulin deficiency and hypertonia secondary to hyperglycemia lead to an incapacity to mobilize high quantities of potassium to the intracellular space [25]. Right ventricular dysfunction can give rise to venous congestion, impaired renal function and secondary hyperkalemia [26]. Lastly, and in contrast to the ES-EORP-HFA registry [12], baseline potassium was found to be the most potent predictor for hyperkalemia development during follow-up.

In regard to the factors independently associated with increased mortality, a number of predictors (male gender, diabetes mellitus, creatinine clearance < 60 mL/min/1.73 m^2^ and advanced NYHA functional class) are constantly identified in both classical studies [27] and in current registries [28]. Anemia is a commonly observed phenomenon in HFrEF and is independently associated with cardiovascular and all-cause mortality [20,29,30,31], and with the presence of ventricular arrhythmias [32]. The association between mortality and right ventricular dysfunction in individuals with HFrEF has been reported previously by Ghio et al. [33]. Lastly, the protective effect observed with ACEI/ARB use is consistent with the data of widely known studies that have marked the evolution of heart failure with reduced ejection fraction. [34,35,36].

Special mention should be made of the finding that normal–high potassium levels (5–5.5 mEq/L) are a protective factor against overall mortality. Classical studies [15] showed that potassium levels of up to 5.5 mEq/L appear to be safe in patients with HFrEF. In this same line, Hoss et al. [19] found that normal–high blood potassium levels were associated with lower all-cause mortality than normal levels. A subanalysis of the DIG trial [16] found no mortality differences between normal and elevated potassium levels. Additionally, the safety of mild hyperkalemia has been evaluated in the context of various mineralocorticoid receptor antagonist (MRA) clinical trials. Thus, a substudy of the RALES trial in patients with chronic heart failure demonstrated that the administration of spironolactone did not result in an increase in mortality rates, despite an observed increase in potassium levels. [37]. In the EMPHASIS-HF clinical trial [38], a greater incidence of renal function deterioration and hyperkalemia was noted in the eplerenone treatment group. However, as long as serum potassium levels did not exceed 5.5 mEq/L, these adverse events did not compromise the favorable prognostic outcomes of the treatment [39]. These findings could be explained because potassium elevation to such values in patients with HFrEF reduces the QT interval (baseline and corrected) and its dispersion [15,40]. In this regard, it has been hypothesized that part of the clinical and prognostic benefits of the treatment with drugs that inhibit the renin–angiotensin–aldosterone system is attributable to the slight increase in potassium levels [34,41], since this reduces the incidence of hypokalemia and hence the risk of sudden cardiac death. In this sense, different studies found that a reduction in potassium concentration (even with normal–low values, 4.1 mEq/L versus 4.4 mEq/L) was an independent predictor of sudden cardiac death in patients with chronic heart failure [42]. The European clinical practice guidelines consider potassium levels up to 5.5 mEq/l to be acceptable and safe. This allows neurohormonal treatments to be titrated or maintained to these values safely and with the prognostic benefits associated with their use [20]. Some studies point to hyperkalemia as a risk marker of suboptimal treatment rather than as a morbidity–mortality risk factor in HFrEF, on the grounds that it prevents the introduction or hinders the up-titration of neurohormonal treatment [43,44]. In this regard, the BIOSTAT-CHF study reported that hyperkalemia was a predictor of suboptimal dosing of ACEI/ARB, rather than an indicator of poor clinical outcomes. Interestingly, the study also found that normal–high levels of potassium did not impede the favorable effects of successful up-titration of these medications [45]. The European ESC-EORP-HFA Heart Failure Long Term Registry initially found both hypo- and hyperkalemia to be associated with poor clinical outcomes; however, after adjusting for the suspension of renin–angiotensin system inhibitors, hyperkalemia was not found to be associated with increased mortality [46]. Overall, these findings suggest that hyperkalemia entails the underdosing or discontinuation of renin–angiotensin–aldosterone system inhibitors, which is associated with poorer clinical outcomes beyond the potential arrhythmogenic effects [43,47]. Based on the findings of our study and of those described above [15,16,19,34,37], and taking into account the recommendations from the European heart failure guidelines [20], normal–high potassium levels (up to 5.5 mEq/L) should make possible to maintain ACEI/ARB/ARNI and MRA treatments under close monitoring, while potassium > 5.5 mEq/L may require dose adjustment or the introduction of the new potassium binders [44], which play an emergent and promising role.

The present investigation is subject to certain limitations. First of all, the present study analyzed a sample consisting of a higher proportion of males (76.6%) as compared to females (23.4%). Nevertheless, this gender distribution is consistent with previous studies conducted in similar settings, which have reported comparable figures ranging from 70% to 78% [12,21,48]. It is also important to mention that the present investigation is a retrospective observational study, and it is important to note that our operational definition of hyperkalemia diverges from the definition employed in clinical practice guidelines and the majority of previous research (potassium > 5.0 mEq/L) [20]. However, as mentioned above, a potassium concentration of 5.5 mEq/L represents the cut-off level considered to maintain neurohormonal treatment unchanged. Likewise, although the usual practice at our unit is to repeat the measurements of altered potassium levels in order to discard pseudohyperkalemia, the retrospective nature of the study precludes us from confirming the universality of this practice in all instances. Finally, the percentage of individuals receiving drugs included in the European heart failure guidelines with a demonstrated effect upon mortality (such as ARNIs and sodium-glucose cotransporter 2 inhibitors) [20] was low, since their use was not stated in the existing guidelines at the beginning of the study [49]. Most of these limitations could be overcome by conducting a prospective study to corroborate the results obtained.

Consequently, the present study can be regarded as a means of generating hypotheses, with the aim of conducting a prospective investigation in the contemporary era, which encompasses all the latest therapeutic interventions that have emerged in the domain of heart failure.

## 5. Conclusions

Hyperkalemia is a common finding in patients with HFrEF with an impact on the optimization of neurohormonal treatment. The closer to the start of follow-up, the higher the risk of developing hyperkalemia. Periodic determinations of serum potassium are needed in all patients presenting with HFrEF, particularly at the beginning of treatment. In our retrospective study, potassium levels in the normal–high range (5–5.5 mEq/L) seem to be safe and are not associated with increased mortality.

## Figures and Tables

**Table 1:** Baseline characteristics of the study population.

Characteristics		N = 1013
Male gender, n (%)		776 (76.6%)
Age (years)		66.6 ± 11.2
Arterial hypertension, n (%)		605 (59.7%)
Diabetes mellitus, n (%)		327 (32.3%)
Smoking habit (former and current habit), n (%)		535 (52.8%)
Alcohol abuse (former and current: >2 alcoholic drink equivalents (per day) in men and >1 in women), n (%)		442 (43.6%)
Body mass index (Kg/m^2^)		28.8 ± 4.8
NYHA functional classification ≥2, n (%)		703 (69.5%)
Systolic hypertension, (mmHg)		130.8 ± 21.4
Diastolic hypertension, (mmHg)		71.7 ± 11.5
Primary etiology, n (%)		
	Ischemic	364 (35.9%)
	Idiopathic	200 (19.7%)
	Alcoholic	113 (11.2%)
	Tachycardia-induced cardiomyopathy	95 (9.4%)
	Hypertensive	94 (9.3%)
	Others	147 (14.5%)
End-diastolic left ventricular diameter, (mm)		59.5 ± 7.8
Left ventricular ejection fraction (%)		33.8 ± 9.1
Mitral valve regurgitation (any grade), n (%)		799 (79.5%)
Right ventricle function—TAPSE (mm)		18.9 ± 4.3
Pulmonary artery systolic pressure (mmHg)		40.9 ± 11.8
Hematocrit (%)		41.3 ± 5.1
Creatinine clearance MDRD (mL/min/1.73 m^2^)		73.1 ± 24.66
Creatinine clearance <60 mL/min/1.73 m^2^, n (%)		296 (29.2%)
Baseline potassium (mEq/L)		4.66 ± 0.44
High normal potassium (5–5.5 mEq/L), n (%)		177 (17.9%)
Potassium >5.5 baseline (mEq/L), n (%)		24 (2.42%)
Treatment (first consultation), n (%)		
	ARNI/ACEI/ARB	803 (79.3%)
	BB	988 (97.5%)
	MRA	592 (58.4%)
	ISGLT2	6 (0.6%)
	Loop diuretics	615 (60.7%)
	Potassium supplements	2(0.2%)
Drug doses at first consultation (over 1, maximum dose in ESC guidelines)		
	ARNI/ACEI/ARB	0.59 ± 0.39
	BB	0.82 ± 0.30
	MRA	0.48 ± 0.14
	Loop diuretic	1.41 ± 0.71
Treatment (at 1 year), n (%)		
	ARNI/ACEI/ARB	965 (97.8%)
	BB	953 (97.3%)
	MRA	560 (70%)
	Loop diuretic	509 (53.9%)
Drug doses at 1 year (over 1, maximum dose in ESC guidelines)		
	ARNI/ACEI/ARB	0.81 ± 0.48
	BB	0.85 ± 0.30
	MRA	0.47 ± 0.16
	Loop diuretic	1.17 ± 0.76
Devices, n (%)		
	IAD	151 (14.9%)
	CRT	19 (1.9%)
	CRT-IAD	63 (6.2%)
Characteristics Electrocardiographic/Holter, n (%)		
	Sinus rhythm	648 (66.3%)
	Atrial fibrillation/flutter	325 (33.3%)
	LBB	229 (27.4%)
	QRS < 120 ms	389 (38.4%)
	Ventricular arrhythmias	207 (20.4%)

Abbreviations: ACEI: angiotensin-converting enzyme inhibitor; ARB: angiotensin II receptor blockers; ARNI: angiotensin receptor neprilysin inhibitor; IAD: implantable automatic defibrillator; ISGLT2: inhibitor of the sodium glucose co-transporter-2; BB: beta-blocker; CRT: cardiac resynchronization therapy, ESC: European Society of Cardiology; LBB: left bundle block; MDRD: modification of diet in renal disease; MRA: mineralocorticoid receptor antagonist; NYHA: New York Heart Association; TAPSE: tricuspid annular plane systolic excursion.

**Table 2:** Predictors of hyperkalemia: univariate analysis.

Variable	*p*	Hazard Ratio	95% Confidence Interval
Age	<0.001	1.04	1.03–1.06
Creatinine clearance MDRD (mL/min/1.73 m^2^)	<0.001	0.98	0.98–0.99
QRS < 120 ms	<0.001	0.48	0.33–0.69
Baseline potassium	<0.001	4.14	2.96–5.79
Hematocrit	0.001	0.95	0.93–0.98
Diabetes mellitus	<0.001	1.64	1.26–2.14
End-diastolic left ventricular diameter	0.032	1.02	1.00–1.04
Pulmonary artery systolic pressure	0.003	1.02	1.01–1.04
Right ventricle function—TAPSE (mm)	0.006	0.95	0.91–0.98
Mitral valve regurgitation (any grade)	0.005	1.56	1.15–2.13
NYHA ≥ 2	0.261	1.36	0.80–2.31
Diastolic blood pressure	<0.001	0.97	0.96–0.99
Beta-blockers	0.045	0.46	0.22–0.98
Loop diuretics	0.021	1.48	1.06–2.07
Arrhythmias	0.195	1.28	0.90–1.82
Device (CRT-IAD)	0.178	1.38	0.87–2.20
Ischemic heart disease	0.034	1.40	1.03–1.92

Abbreviations: CRT-IAD: cardiac resynchronization therapy—implantable automatic defibrillator, MDRD: Modification of diet in renal disease; NYHA: New York Heart Association; TAPSE: Tricuspid annular plane systolic excursion.

**Table 3:** Predictors of hyperkalemia: multivariate analysis.

Variable	*p*	Hazard Ratio	95% Confidence Interval
Baseline potassium	<0.001	3.13	2.15–4.60
Creatinine clearance MDRD (mL/min/1.73 m^2^)	0.013	0.99	0.98–0.99
Right ventricle function—TAPSE (mm)	0.016	0.95	0.91–0.99
Diabetes mellitus	0.047	1.40	1.01–1.96

Abbreviations: MDRD: Modification of diet in renal disease, TAPSE: Tricuspid annular plane systolic excursion.

**Table 4:** Predictors of overall mortality: univariate analysis.

Variable	*p*	Hazard Ratio	95% Confidence Interval
Female gender	<0.001	0.52	0.35–0.77
End-diastolic left ventricular diameter	<0.001	1.03	1.01–1.05
Pulmonary artery systolic pressure	<0.001	1.03	1.01–1.04
Right ventricle function–TAPSE (mm)	<0.001	0.93	0.90–0.97
Arterial hypertension	0.011	1.47	1.09–1.97
Diabetes mellitus	<0.001	1.57	1.23–1.99
NYHA (per each grade)	<0.001	2.02	1.34–3.00
Age	<0.001	1.04	1.03–1.06
Smoking	0.001	1.27	1.10–1.47
Creatinine clearance MDRD (mL/min/1.73 m^2^)	<0.001	0.98	0.97–0.99
Arrhythmias	<0.001	1.91	1.41–2.59
Baseline potassium	<0.001	1.13	1.07–1.19
Hematocrit	0.001	0.95	0.97–0.98
Systolic blood pressure	0.029	0.99	0.98–0.99
Diastolic blood pressure	0.002	0.98	0.97–0.99
ACEI/ARB	<0.001	0.28	0.16–0.50
Loop diuretics	<0.001	2.18	1.58–3.02
Ischemic heart disease	<0.001	1.67	1.26–2.21
CRT-IAD start	0.049	1.51	1.00–2.28

Abbreviations: ACEI: angiotensin-converting enzyme inhibitor; ARB: angiotensin II receptor blockers; CRT-IAD: cardiac resynchronization therapy—implantable automatic defibrillator, MDRD: modification of diet in renal disease; NYHA: New York Heart Association; TAPSE: tricuspid annular plane systolic excursion.

**Table 5:** Predictors of overall mortality: multivariate analysis.

Variable	*p*	Hazard Ratio	95% Confidence Interval
Age	0.002	1.03	1.01–1.05
Female gender	<0.001	0.43	0.27–0.68
Diabetes mellitus	0.003	1.64	1.19–2.27
NYHA (per each grade)	0.034	1.58	1.04–2.42
Hematocrit	0.013	0.96	0.94–0.99
Creatinine clearance ≥ 60 mL/min/1.73 m^2^(MDRD)	0.007	0.61	0.43–0.48
Mild hyperkalemia (5–5.5)	0.025	0.60	0.38–0.94
Right ventricle function—TAPSE (mm)	0.012	0.95	0.92–0.99
Ventricular arrhythmia	<0.001	1.85	1.32–2.59
ACEI or ARB	0.020	0.36	0.16–0.85

Abbreviations: ACEI: angiotensin-converting enzyme inhibitor; ARB: angiotensin II receptor antagonist; MDRD: modification of diet in renal disease; NYHA: New York Heart Association; TAPSE: tricuspid annular plane systolic excursion.

## Data Availability

Data will be available after reasonable request to the corresponding author.
